# Surround suppression and sparse coding in visual and barrel cortices

**DOI:** 10.3389/fncir.2012.00043

**Published:** 2012-07-05

**Authors:** Robert N. S. Sachdev, Matthew R. Krause, James A. Mazer

**Affiliations:** ^1^Department of Neurobiology, Yale School of Medicine, New HavenCT, USA; ^2^Department of Psychology, Yale University, New HavenCT, USA

**Keywords:** inhibition, S1, somatosensory cortex, sparse coding, suppression, V1, vibrissae, visual cortex

## Abstract

During natural vision the entire retina is stimulated. Likewise, during natural tactile behaviors, spatially extensive regions of the somatosensory surface are co-activated. The large spatial extent of naturalistic stimulation means that surround suppression, a phenomenon whose neural mechanisms remain a matter of debate, must arise during natural behavior. To identify common neural motifs that might instantiate surround suppression across modalities, we review models of surround suppression and compare the evidence supporting the competing ideas that surround suppression has either cortical or sub-cortical origins in visual and barrel cortex. In the visual system there is general agreement lateral inhibitory mechanisms contribute to surround suppression, but little direct experimental evidence that intracortical inhibition plays a major role. Two intracellular recording studies of V1, one using naturalistic stimuli (Haider et al., 2010), the other sinusoidal gratings (Ozeki et al., 2009), sought to identify the causes of reduced activity in V1 with increasing stimulus size, a hallmark of surround suppression. The former attributed this effect to increased inhibition, the latter to largely balanced withdrawal of excitation and inhibition. In rodent primary somatosensory barrel cortex, multi-whisker responses are generally weaker than single whisker responses, suggesting multi-whisker stimulation engages similar surround suppressive mechanisms. The origins of suppression in S1 remain elusive: studies have implicated brainstem lateral/internuclear interactions and both thalamic and cortical inhibition. Although the anatomical organization and instantiation of surround suppression in the visual and somatosensory systems differ, we consider the idea that one common function of surround suppression, in both modalities, is to remove the statistical redundancies associated with natural stimuli by increasing the sparseness or selectivity of sensory responses.

## Introduction

In retinal ganglion cells, the response to a small stimulus of the appropriate luminance polarity (light or dark) positioned in the center portion of the receptive field is larger than the response evoked by a larger stimulus of the same polarity extending beyond the center. Similarly, in the rodent whisker system, single whisker stimulation typically evokes larger responses than stimulation of two or more whiskers. At first glance, results like these seem puzzling because they suggest that spatially extensive and therefore more naturalistic stimuli that engage the entire eye or multiple whiskers are somehow less effective at driving sensory responses than spatially restricted stimuli.

**Figure 1:**
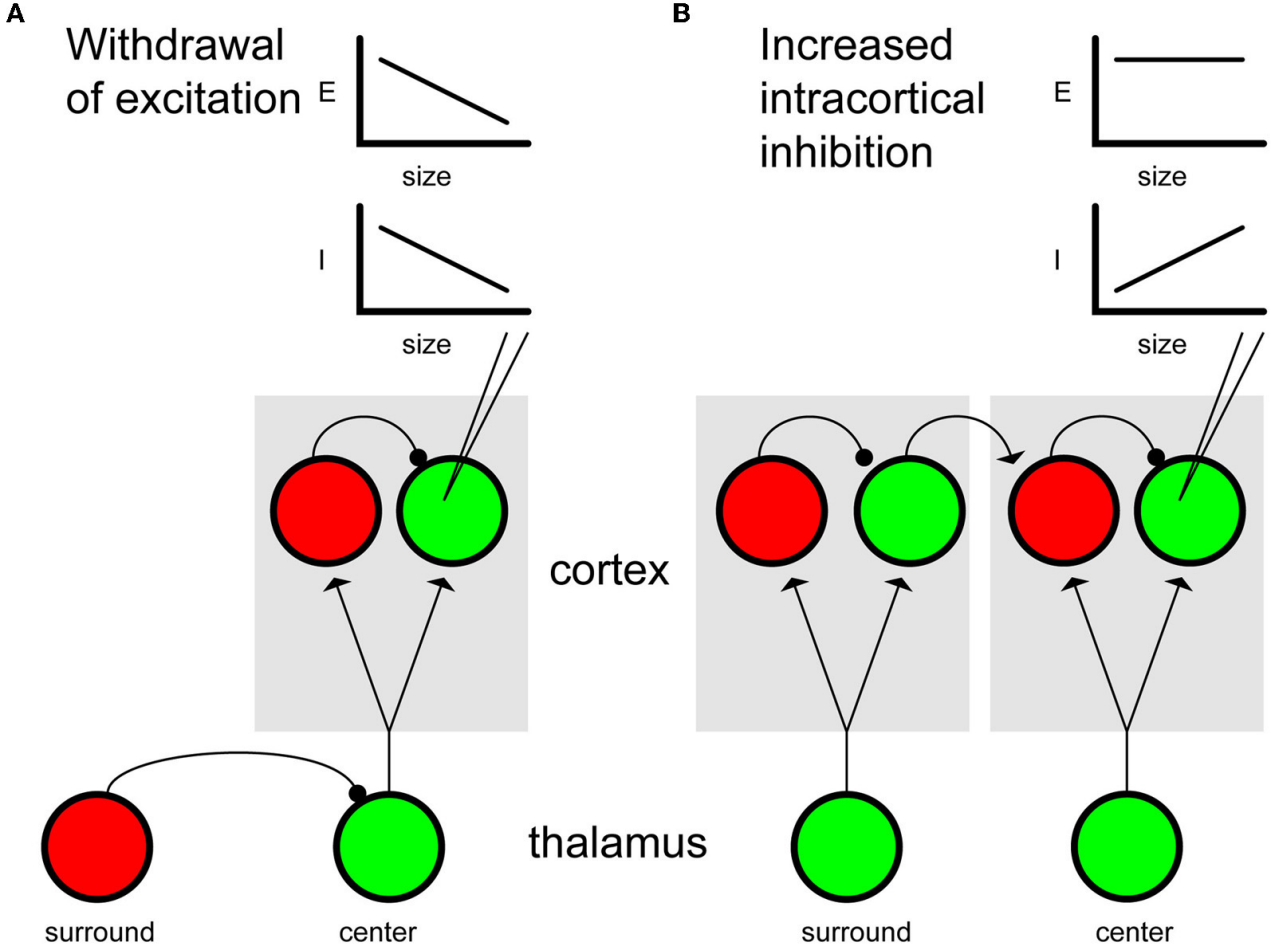
**Mechanisms of surround suppression. (A)** Withdrawal of excitation. During center-only stimulation, thalamocortical feed-forward projections target both excitatory (green) and inhibitory (red) cortical neurons, leading to both excitation and inhibition. Increasing the spatial extent of the stimulus recruits inhibitory intrathalamic connections resulting in a net reduction of feed-forward drive to cortex. Because drive is reduced to both pyramidal cells and interneurons, withdrawal of excitation ultimately leads to a balanced decrease in both excitatory (E) and inhibitory (I) conductance in pyramidal neurons (inset plots represent conductances recorded from the pyramidal neuron indicated by electrode). Although withdrawal of thalamocortical excitation is illustrated here, the same model could apply to excitatory feedback projections from higher cortical areas. **(B)** Intracortical inhibition. Spatially extensive stimuli that activate the surround region (left column) engage local interneurons representing the CRF (right column) via long-range excitatory connections. When the surround is activated, indirect activation of local interneurons leads to increased inhibitory conductances in pyramidal neurons, while excitatory conductances remain constant (see inset plots).

To address this apparent conundrum, we review some of the different types of neuronal circuits that have been proposed to generate surround suppression in the visual and somatosensory systems. We begin by defining some basic terminology. The terms suppression and inhibition are often used interchangeably, however, in the following discussion we distinguish between the two by defining suppression as a reduction in firing rate and inhibition as the process of eliciting inhibitory post synaptic potentials (IPSPs), presumably by presynaptic GABA release. This distinction is critical because the *phenomenon* of suppression can be driven by a number of different *mechanisms* including inhibition, but also withdrawal of excitation or even changes in intrinsic cell properties (see Figure 1). We will argue that in the visual system an important function of surround suppression is to increase the response sparseness of cortical neurons in order to eliminate the redundancies that are a hallmark of natural visual stimuli (Field, 1987). The term sparseness is often used loosely in the literature, but here it is important to define exactly what we mean by sparseness. Sparseness is a property of the distribution of neuronal firing rates; Rolls and Tovee (1995) defined sparseness as a measure of the kurtosis or shape of the firing rate distribution. A recent article by Willmore and colleagues (2011) defined the types of response sparseness that will be discussed here. “Lifetime sparseness” is a property of single neurons and reflects the shape or peakedness of a neuron's firing rate distribution in response to a fixed set of stimuli. Neurons with high lifetime sparseness are highly selective—they are generally silent but respond vigorously to a small fraction of stimuli. In contrast, “population sparseness” is a property of a neuronal ensemble and reflects the fraction of neurons in the ensemble that respond to a given stimulus. In brain regions with high population sparseness, only a small fraction of neurons are activated in response to a given stimulus. Finally, a third common usage of sparseness refers to any neuron or neuronal ensemble that exhibits a low mean firing rate. In the visual system literature, the first two usages are most common, depending on whether the data arises from single neuron or neuronal population recordings. In the somatosensory system, the third usage is most common. It is important to note that measurements of the first two types of sparseness depend on both neurons and stimuli. Low mean rate sparseness, however, may or may not be dependent on the stimuli. In fact, some studies (e.g., Brecht and Sakmann, 2002b; Petersen, 2002, 2003) have used the term “sparse” to describe observed low mean spontaneous rates in S1. When response sparseness is studied in barrel cortex, it is common to quantify sparseness based on the mean firing rate in response to many repetitions of a single stimulus (Brecht and Sakmann, 2002a; Brecht et al., 2003; Jadhav et al., 2009). To be clear, sparse coding and sparse activity are not the same thing. The former is a property of the neural encoding of sensory stimuli, while the latter may be indicative of certain network architectures, it has no direct relationship to stimulus encoding (see Willmore et al. (2011) for a complete discussion).

Note that increased response sparseness is often associated with a reduction in spiking activity, either driven or spontaneous, although this is not necessarily the case (Willmore et al., 2011). This reduction corresponds to suppression, as defined above. Although there is substantial evidence that center-surround interactions contribute to sparsificiation (Vinje and Gallant, 2000; Haider et al., 2010), it is generally an open question as to whether the observed suppression is mediated by inhibition. Interestingly, recent studies of neurons in barrel cortex demonstrate that spatially extensive stimuli that engage surround mechanisms can increase selectivity (Jacob et al., 2008), however, they can also either reduce or increase the precision of the response (Webber and Stanley, 2006). Unfortunately it is difficult to apply exactly the same classes of stimuli to both visual and somatosensory whisker systems (Jacob et al., 2008), and thus it is difficult to disambiguate the effects of stimulus differences from differences in neural circuitry. Future studies will be required to determine whether spatially extensive, naturalistic stimuli increase selectivity in both visual and barrel cortex.

In the early visual system, surround suppression appears to be ubiquitous. At least one type of surround suppression, lateral inhibition, arises as early as the retina [limulus: (Hartline et al., 1956), frog: (Barlow, 1953); cat: (Kuffler, 1953)]. It has been more than 70 years since Hartline demonstrated that simultaneous stimulation of adjacent ommatidia in the limulus compound eye suppressed firing rates relative to single ommatidium stimulation (Hartline, 1942). Throughout the 1950s, studies by Hartline et al., reported similar suppressive effects in vertebrate retinal ganglion cells. These seminal studies, which were among the first to describe and then carefully map the spatial-opponent organization of retinal ganglion cells, suggested a functional role for surround suppression in the visual system, namely to enhance sensitivity to contrast edges and effectively reduce, or even eliminate, responses to constant illumination and other redundant information in the visual environment. Similar lateral interactions are thought to enhance the precision of other sensory representations: two point somatosensory discrimination thresholds (Von Békésy, 1967; Mountcastle, 1975; Laskin and Spencer, 1979), frequency discrimination thresholds in the auditory system (Suga and Manabe, 1982; Calford and Semple, 1995; Sutter and Loftus, 2003) and odor discrimination thresholds in rodents (Rall et al., 1966; Yokoi et al., 1995; Phillips et al., 2012). At this time, our understanding of the neural mechanisms underlying surround suppression is arguably most complete in the mammalian visual and somatosensory systems. However, even in these relatively well-studied systems, the exact role of inhibition, as defined above, remains poorly understood, a problem perhaps exacerbated by inconsistent nomenclature for both mechanisms and phenomena. Here we shall consider several possible circuit-level mechanisms proposed to account for the surround suppression observed in visual and barrel cortex, including synaptic inhibition.

## Receptive field organization in the early visual system

Retinal ganglion cells have concentrically organized spatial receptive fields with either ON or OFF centers. Light increments falling in the center of an ON cell's receptive field increase firing while light increments in the surround reduce firing and *vice versa* for OFF center cells (Hartline, 1938; Kuffler, 1953; Hubel and Wiesel, 1962). When the retinal ganglion cells are stimulated with a broad, uniform luminance field covering both center and surround, center and surround responses cancel (Kuffler, 1953). This center-surround interaction means that retinal ganglion cells effectively signal the difference in the illumination between the center and surround. This center-surround opponent structure is reiterated in the visual thalamus, where receptive fields in the dorsal lateral geniculate nucleus (LGN) also have a center-surround structure thought to arise directly from retinogeniculate inputs (Hubel and Wiesel, 1961). While LGN neurons receive some cortico-thalamic input from layer VI neurons in primary visual cortex (V1 or area 17), these inputs are thought to be largely modulatory, relatively weak, and contribute little to receptive field structure in the LGN (Sherman and Guillery, 1998; but see Olsen et al., 2012). Receptive field organization undergoes a substantial change between the LGN and primary visual cortex, where neurons exhibit emergent selectivity for novel stimulus properties, like orientation, derived, at least in part, from the convergence of thalamocortical inputs (Hubel and Wiesel, 1962). While V1 neurons do not have the same simple, circularly symmetric center-surround receptive field structures seen in the retina and LGN, they often have an opponent organization with center and surround regions, where, generally speaking, stimulation of the center is facilitatory and stimulation of the surround is suppressive. The classical receptive field (CRF) in V1 is generally defined as the region where the onset or offset of isolated stimuli elicit a change in firing rate. This is in contrast to the non-classical receptive field (nCRF) where isolated stimuli have no effect on firing, but when paired with CRF stimuli, nCRF stimulation can modulate responses to the CRF stimulus. The nCRF can extend well beyond the CRF (Angelucci and Sainsbury, 2006). It is important to note that during natural vision, where stimuli engage the entire retina at once, it is likely that the entire early visual system operates in a regime where lateral interactions and surround suppression are important, or even dominant.

One long-standing and highly influential idea is that the nCRF or surround provides “context” for stimuli appearing in the CRF, enhancing the ability of neurons to detect or discriminate orientation and motion discontinuities, textures, and contour curvature (Blakemore and Tobin, 1972; Nelson and Frost, 1978; Allman et al., 1985; Gilbert and Wiesel, 1990; Levitt and Lund, 1997; Walker et al., 1999; Bakin et al., 2000) or even facilitate high level target selection via pop-out mechanisms (Knierim and Van Essen, 1992). In V1, many of these context-dependent responses require integration of visual signals arising from beyond the CRF and therefore must depend on lateral interactions. A full understanding of surround suppression requires identification and characterization of the neural circuits that underlie these lateral interactions.

Recently, an alternative, or perhaps complementary, framework for considering center-surround interactions in V1 has arisen as based on theoretical ideas about efficient neural coding of natural stimuli (Barlow, 1972; Van Hateren, 1992; Olshausen and Field, 1996; Attwell and Laughlin, 2001). These studies noted that natural visual stimulation is highly redundant, in both time and space. Identification and elimination of these naturally occurring spatial and temporal correlations allows the neural code to transmit information more efficiently. Van Hateren (1992) and Olshausen and Field (1996) described biologically plausible neural networks that could perform this type of decorrelation based on lateral or mutual inhibition. One of the first experiments to test this idea was a study by Vinje and Gallant (2000) that reported simultaneous activation of the CRF and nCRF using highly dynamic, naturalistic stimuli resulted in non-linear changes in visual selectivity that ultimately led to a net increase in response sparseness, where response sparseness serves as a proxy for neural selectivity (Rolls and Tovee, 1995; Olshausen and Field, 2004; Lehky et al., 2005; Yao et al., 2007; Yen et al., 2007; Tolhurst et al., 2009). Increased response sparseness means that each spike transmits more information about the stimulus (Vinje and Gallant, 2002). Because these studies relied on extracelluar recording techniques, they were unable to shed light on the nature of the neuronal circuits and biophysical mechanisms that underlie the sparse neuronal code. Specifically, they were unable to address the question of whether the sparse, efficient code is instantiated by inhibitory mechanisms or simply reflects a withdrawal of excitation. If inhibition is involved, where does it come from?

There are several plausible circuit mechanisms that could result in surround suppression and instantiate a sparse neural code. Suppression could arise subcortically, intracortically or even via feedback connections from extrastriate areas (see Figure 1). Two classes of models for generating surround suppression in V1 have been discussed recently in the literature. In one model intracortical inhibition, driven by differential activation of inhibitory interneurons intrinsic to V1, generates surround suppression (Figure 1B). The alternative model is based on a withdrawal of excitation and is generally thought to be a withdrawal of feedfoward thalamocortical excitation, but could also apply to feedback projections from higher cortical areas back to V1 (Figure 1A). Here we list some of the experimental findings in support of the former hypothesis:

1. Since V1 is retinotopically organized, the cortical representations of the center and surround regions are always in close anatomical proximity, well within the distance limits imposed by the length of horizontal and interneuron projections. Indirect intracortical inhibition via long-range horizontal connections appears to be sufficient to account for many of the observed surround suppressive effects (Rockland and Lund, 1983; Gilbert et al., 1996; Somers et al., 1998). There are extensive local axon collaterals of layer II/III and layer V pyramidal neurons that offer a path for surround suppression (Gilbert and Wiesel, 1983; Allman et al., 1985; Gilbert and Wiesel, 1990; Born and Tootell, 1991; McGuire et al., 1991; Gilbert and Wiesel, 1992; Knierim and Van Essen, 1992; Deangelis et al., 1994; Toth et al., 1996; Nothdurft et al., 1999; Anderson et al., 2000; Dragoi and Sur, 2000; Fitzpatrick, 2000; Hupe et al., 2001; Stettler et al., 2002). However, it is also possible that surround suppression arises from intracortical inhibition activated by feedback connections from extrastriate areas (Bair et al., 2003; Angelucci and Sainsbury, 2006). Extrastriate feedback projections make modulatory connections (Rockland and Pandya, 1979; Felleman and Van Essen, 1991). These connections, like the intracortical horizontal connections in V1, are exclusively excitatory, so any inhibitory effects arising from feedback projections must depend on local activation of inhibitory interneurons in V1.
2. *In vivo* voltage-sensitive dye imaging has demonstrated that the activity evoked by spatially restricted stimuli in V1 can undergo extensive horizontal spread. A small visual stimulus can evoke a wave of depolarization in monkey visual cortex that travels at 0.2–0.5 m/s and covers a region 10 times larger than the zone of initial activation (Grinvald et al., 1994; Benucci et al., 2007). Similar traveling waves have been described in the cat and rat (Girard et al., 2001; Slovin et al., 2002; Benucci et al., 2007; Xu et al., 2007). The original observations of traveling waves in V1 (Grinvald et al., 1994) reported relatively low propagation rates, too slow to account for surround suppression effects, supporting the idea that suppression arises from extrastriate feedback (Bair et al., 2003; Angelucci and Sainsbury, 2006). However, more recent studies, perhaps due to improved methods and newer dyes, have reported faster propagation rates fully capable of supporting surround suppression (Benucci et al., 2007). Imaging studies provide direct physiological evidence of a fast, responsive, and extensive intracortical network capable of supporting the lateral connectivity required for surround suppression. However, the final step of connecting this depolarizing wave to local inhibitory interneurons has yet to be made.
3. Many surround suppressive phenomena, including length selectivity and texture popout, are highly orientation selective, consistent with a cortical origin (Knierim and Van Essen, 1992; Deangelis et al., 1994; Li and Li, 1994; Jones et al., 2001; Angelucci et al., 2002; Cavanaugh et al., 2002; Bair et al., 2003; Webb et al., 2005; Ozeki et al., 2009).

However, it is important to note that not all suppressive phenomena in V1 are orientation selective or necessarily involve intracortical inhibition. Many studies suggest that cross-orientation suppression has non-cortical origins (Morrone et al., 1987; Freeman et al., 2002; Priebe and Ferster, 2006; Katzner et al., 2009). For example, Priebe and Ferster (2006) recorded intracellular synaptic potentials in response to stimuli that evoked cross-orientation suppression and found reductions in both excitatory and inhibitory contributions, consistent with a withdrawal of thalamocortical excitation (e.g., Figure 1A).

As noted above, long range connections, be they feedforward, intracortical or feedback, are overwhelmingly excitatory. For these connections to drive synaptic inhibition, they must act through local inhibitory interneurons. In the context of the canonical neocortical circuit (Wilson and Cowan, 1973; Douglas and Martin, 1994; Douglas et al., 1995), lateral interactions resulting in surround suppression are somewhat hard to explain. In the canonical model, sensory stimuli activate thalamocortical projection neurons, which in turn simultaneously activate both excitatory and inhibitory neurons in primary visual cortex. The excitatory cells in turn make both recurrent and long-range synaptic connections. Recurrent, reciprocal connections between excitatory neurons can amplify excitatory activity, leading to increased excitatory drive for both local and distant inhibitory neurons via horizontal connections. At the same time, inhibitory neurons locally inhibit both excitatory and other inhibitory neurons. In this “standard” model, excitation balances inhibition (Shu et al., 2003; Wehr and Zador, 2003; Haider et al., 2006; Atallah and Scanziani, 2009): whenever activity increases in excitatory neurons, activation of inhibitory neurons increases in lockstep, keeping the balance. Conversely, when inhibitory neurons are activated, they suppress nearby excitatory neurons, leading to a reduction in both excitation and inhibition. Again, in the model, balance is maintained.

One thing the canonical model does not take into account is the fine temporal structure of synaptic inputs. Superimposed on any sustained depolarization due to recurrent activity are membrane potential fluctuations generated by synaptic barrages that vary in amplitude and frequency (Cowan and Wilson, 1994; Pare et al., 1998; Steriade et al., 2001; Timofeev et al., 2001; Hasenstaub et al., 2005; Rudolph et al., 2007; Cardin et al., 2009; Sohal et al., 2009; Tiesinga and Sejnowski, 2009). These fluctuations are dominated by inhibition at frequencies between approximately 2 and 100 Hz (Cowan and Wilson, 1994; Hasenstaub et al., 2005) and the membrane potential of single neurons is dominated by inhibition (Rudolph et al., 2007). Inhibitory networks control the amplitude, extent, and duration of activation of recurrent excitatory cortical networks, but also the precise timing of action potentials and thus, network synchronization (Rudolph et al., 2007; Cardin et al., 2009; Sohal et al., 2009).

The canonical model also largely ignores the diversity of inhibitory neurons. Some inhibitory neurons innervate pyramidal cell bodies and proximal dendrites, while others target the axon initial segment (Cobb et al., 1995; Miles et al., 1996; Pouille and Scanziani, 2001; Buzsaki et al., 2004; Rudy et al., 2011), still others are electrotonically coupled through gap junctions to other cortical GABAergic neurons (Galarreta and Hestrin, 1999; Gibson et al., 1999; Hormuzdi et al., 2001; Galarreta and Hestrin, 2002).

For intracortical inhibition to be effective in generating surround suppression, inhibition must be temporarily uncoupled from excitation to cause a withdrawal of excitation. Some interneurons fire in phase with excitatory cortical neurons, while others fire out of phase with the bulk of excitatory and inhibitory neurons in cortex (Crochet and Petersen, 2006; Gentet et al., 2010). Intracellular recordings show that under some circumstances inhibitory conductances can dominate or be differentially activated during sensory stimulation (Timofeev et al., 2001; Monier et al., 2003; Rudolph et al., 2007; Haider et al., 2010). The model also simplifies the complex patterns of laminar interactions that have been described in the literature. For example, the effects of layer II/III pyramidal cell stimulation depend on recording site: layer II/III pyramidal neurons in neighboring columns are suppressed while layer V pyramidal cells in the same column are facilitated and neurons in layers IV and VI are relatively unaffected (Adesnik and Scanziani, 2010).

### Surround suppression and inhibition in visual cortex

If spatial surround suppression is driven by increased intracortical inhibition, whether locally or by feedback connections, it follows that: (1) stimuli that elicit surround suppression should increase inhibition, i.e., engaging the surround should lead to an increase in the amplitude or frequency of inhibitory post-synaptic potentials, (2) inhibitory interneurons should show surround facilitation, not surround suppression, (3) inhibitory interneurons should have larger spatial receptive fields than their target pyramidal neurons, and (4) excitatory pyramidal cells with receptive field centers near the edges of the stimuli, that would provide the excitatory drive to local interneurons, should exhibit elevated firing rates.

Haider and colleagues (2010) measured intracellular responses of V1 neurons in the paralyzed, anesthetized cat to naturalistic movie stimuli, vignetted to engage either the CRF alone or both the CRF and nCRF simultaneously. They directly measured excitatory and inhibitory postsynaptic potentials evoked by the small and large stimuli in identified classical regular spiking pyramidal cells and fast spiking interneurons. In regular spiking pyramidal neurons, they found that simultaneous stimulation of the CRF and nCRF led to significant enhancement of IPSP amplitudes during specific portions of natural stimuli, with an overall increase in the amplitude of inhibitory post-synaptic potentials (~40%), relative to CRF stimulation. No significant changes in the overall amplitudes of the excitatory post-synaptic potentials were observed in response to the same stimuli. There was, however, a significant increase in the temporal precision and trial-to-trial reliability of both evoked excitatory potentials and the spiking response when the nCRF was stimulated. The net result of nCRF/surround stimulation was a sparser stimulus response, presumably reflecting a non-linear increase in visual selectivity. In contrast, recordings from fast spiking interneurons showed exactly the opposite effect: simultaneous CRF + nCRF stimulation increased firing rates and decreased response sparseness. Finally, Haider et al. (2010) reported that the receptive fields of fast spiking interneurons were systematically larger than those of excitatory regular spiking neurons recorded at similar eccentricities. Together, these results suggest that the increased IPSPs recorded in pyramidal neurons during CRF + nCRF stimulation are in large part generated by fast spiking interneurons.

Ozeki and colleagues (2009) also measured excitatory and inhibitory synaptic potentials evoked by center-only and simultaneous center-surround stimulation using drifting sinusoidal grating stimuli. They reported that surround stimulation reduced both excitatory and inhibitory conductances, leading in a net reduction in mean firing rate to larger stimuli. They also noted a transient (30–50 ms) increase in inhibition evoked by center-surround stimulation in a subset of cells. The authors proposed this transient increase in inhibition was responsible for the subsequent coupled reduction of excitatory and inhibitory conductance, which in turn, led to a net reduction in firing rate (Ozeki et al., 2009). To rule out the possibility that suppression of LGN activity via corticothalamic projections resulted in a withdrawal of excitation, they also recorded responses of LGN neurons to the same stimuli and found no differences between center-only and center-surround responses.

One potentially important difference between these two studies is the spectrotemporal content of the stimuli. Haider and colleagues (2010) used broadband naturalistic movie stimuli, while Ozeki and colleagues (2009) used more traditional drifting sinusoidal grating stimuli. The grating stimuli, while dynamic, are spectrotemporally narrowband, i.e., each stimulus is defined by a single orientation, spatial, and temporal frequency. It is possible these different stimulus classes engage the cortical network in different ways, perhaps placing the local cortical network in different operating regimes. This question currently remains an important topic for future studies.

Both of these studies, however, support the idea that surround suppression in V1 is not a purely thalamocortical mechanism, but rather one that can depend on local intracortical inhibition. Both studies provide evidence that when spatially extended stimuli engage the surround region, there is either a sustained or transient increase in intracortical inhibition that ultimately leads to a reduction in firing rate and, in the case of naturalistic stimulation, an increase in response sparseness. The specific origin of the long-range excitatory signals that drive inhibitory interneurons during surround stimulation has yet to be nailed down. These signals could originate within V1, perhaps even from specific laminae and cell types, or they could originate from extrastriate areas. Additional experiments are required to resolve these questions.

## Whisker to barrel cortex system

### Comparing visual and whisker systems

The rodent somatosensory vibrissal system is very different from the visual system. Moreover, the manner in which these two systems have been studied to date is also very different. First, in the vibrissal system, the representation of the sensory space is discrete and organized around individual whiskers, from the periphery through primary somatosensory cortex. The visual system, with its dramatically higher receptor count, instantiates a more continuous representation of the visual environment at the periphery but discrete sampling and columnar organization does not arise until V1. Second, in the vibrissal system, there is no established notion of classical and non-classical or surround receptive fields. The nCRF, where stimuli that evoke no increase or decrease in firing rate but interact with stimuli delivered to the center receptive field, is rarely studied. The most commonly investigated surround interactions in the vibrissal system are responses to a center whisker and the adjacent surround whiskers, where stimulation of either the center or a surround whisker can evoke increases in firing rate. In contrast to visual cortex, where the CRF is defined as the retinal area where stimuli of the right orientation, size, and luminance can elicit or suppress spikes, in barrel cortex the center receptive field is defined by the single whisker that elicits the shortest latency, largest magnitude response. The surround receptive field includes all the whiskers that evoke longer latency, weaker responses. Aside from the trigeminal ganglion cells, which are directly connected to a single whisker follicle, virtually all neurons in the vibrissal system, from brainstem to barrel cortex, respond to individual stimulation of more than one whisker.

Another major difference between the visual and whisker system is that we have a reasonably well-established intuitive, idea of what constitutes a natural visual stimulus, while the same cannot be said for whisker stimulation. The natural, and therefore ethologically relevant, stimulus set could be single whiskers making discrete contacts during object localization, multiple whiskers making synchronous or asynchronous contact, high frequency slip-stick synchronous or asynchronous movements, or active or passive movement of single or multiple whiskers (Carvell and Simons, 1990; Sachdev et al., 2001, 2002; Hartmann et al., 2003; Knutsen et al., 2005, 2006; Mehta et al., 2007; Mitchinson et al., 2007; Lottem and Azouz, 2008; Ritt et al., 2008; Curtis and Kleinfeld, 2009; Grant et al., 2009; Jadhav et al., 2009; Lottem and Azouz, 2009; O'Connor et al., 2010a). During all these behaviors, whiskers move in different patterns, to different extents, and at different frequencies and velocities, resulting in different patterns of cortical activation.

Another important difference between these two systems is the emergence of novel tuning properties. To date, no emergent property, analogous to orientation tuning in V1, has been identified in barrel cortex—all of the identified tuning properties of barrel cortex neurons, including direction selectivity, are already present in brainstem and thalamic vibrissal neurons. Trigeminal ganglion cells respond to minute deflections of single whiskers (Gibson and Welker, 1983; Lichtenstein et al., 1990; Lottem and Azouz, 2008); ganglion cells discharge during whisker movement or when whiskers contact an object (Leiser and Moxon, 2007). Thalamic neurons and neurons in barrel cortex both exhibit increased activity during both whisking and object contact (Fee et al., 1997; Hentschke et al., 2006; Curtis and Kleinfeld, 2009; De Kock and Sakmann, 2009; Gentet et al., 2010; Crochet et al., 2011; Hill et al., 2011; Huber et al., 2012). The activity of both first order ganglion cells and cortical neurons encode the occurrence of high velocity stick-slip events (Carvell and Simons, 1990; Arabzadeh et al., 2003; Andermann et al., 2004; Mitchinson et al., 2007; Ritt et al., 2008; Curtis and Kleinfeld, 2009; Lottem and Azouz, 2009). Thalamic and cortical responses are not identical: cortical neurons adapt to high frequency stimulation, while thalamic neurons accurately follow high frequency stimuli; thalamic neurons also have more selective angular tuning, respond more vigorously to stimuli, and are more likely to be slowly adapting than the cortical neurons (Simons, 1978; Simons and Carvell, 1989; Ahissar et al., 2001; Sosnik et al., 2001; Kleinfeld et al., 2002; Katz et al., 2006; Melzer et al., 2006). However, it seems like these differences reflect incremental changes, compared to the dramatic emergence of orientation tuning in V1.

### Receptive fields in the whisker to cortex pathway

There are two main pathways by which sensory information from the whiskers can reach barrel cortex. The lemniscal pathway arises from the principal trigeminal nucleus and synapses in the barreloids of the ventral posterior medial nucleus of the thalamus (VPM). These neurons primarily target layer IV barrel cells (Koralek et al., 1988; Chmielowska et al., 1989; Lu and Lin, 1993) and drive the layer IV spiny neurons, which then project to layer II/ III above the barrel or septum (Kim and Ebner, 1999; Petersen and Sakmann, 2001; Feldmeyer et al., 2002; Lubke et al., 2003; Shepherd et al., 2003; Staiger et al., 2004). Layer II/III projections provide a pathway for long-range intracortical connections in barrel-field (Thomson and Bannister, 1998; Reyes and Sakmann, 1999; Holmgren et al., 2003; Feldmeyer et al., 2006; Bruno et al., 2009). Neurons in the lemniscal pathway (principal trigeminal nucleus to VPM thalamus to barrels and layers II/III and V) respond vigorously to the deflection of one whisker (the principal whisker) and respond weakly to deflection of surrounding whiskers (Simons and Carvell, 1989; Armstrong-James et al., 1991; Chiaia et al., 1991; Diamond et al., 1992; Nicolelis and Chapin, 1994; Friedberg et al., 1999; Minnery et al., 2003; Minnery and Simons, 2003).

The paralemniscal pathway arises from the large receptive field cells in the spinal trigeminal complex, projects primarily to the posterior group of thalamus, and terminates in the dysgranular zone around, above, and below each barrel in primary somatosensory cortex. Neurons in the paralemniscal pathway respond equally to the deflection of several whiskers (Woolston et al., 1982; Jacquin et al., 1986, 1989; Chiaia et al., 1991; Diamond et al., 1992; Veinante et al., 2000).

In theory, intracortical lateral interactions could be the source of responses from the surround portion of the receptive field in barrel cortex. However, experiments show that both the center and surround have a substantial sub-cortical origin. The receptive fields of neurons in the principal trigeminal nucleus and ventral posterior thalamic nucleus (lemniscal pathway) shrink to a single whisker after lesions of the spinal trigeminal nucleus, indicating that surround receptive fields are constructed in the brainstem and relayed to thalamus (Lee et al., 1994; Friedberg et al., 1999; Lavallee and Deschenes, 2004; Timofeeva et al., 2004; Kwegyir-Afful et al., 2005). There are also identified anatomical pathways within the thalamus that could support additional lateral interactions. For example, the dendritic arbors of thalamocortical projection neurons can span multiple barreloids, providing a possible anatomical substrate for multi-whisker interactions (Varga et al., 2002; Lavallee and Deschenes, 2004). Like other thalamocortical neurons, these neurons respond to multiple whiskers, but the suppressive interactions mediated by reticular thalamic inhibitory feedback are restricted to the whiskers associated with the barreloids occupied by the neuron's dendrites. Current source density analysis suggests that thalamic inputs mediate a large portion of the multi-whisker receptive field in any given barrel (Roy et al., 2011). Together these studies indicate that the center-surround organization of receptive fields in barrel cortex is largely, or even completely, generated by thalamic input with relatively little intracortical contribution, but note that the detailed receptive field structure depends on the anesthetic state of the animal (Armstrong-James et al., 1991; Goldreich et al., 1999; Fox et al., 2003; Wright and Fox, 2010; Constantinople and Bruno, 2011). However, there is evidence that some lateral interactions, like cross-whisker adaptation, are mediated by intracortical mechanisms (Katz et al., 2006). Just as in visual cortex, there is the potential for intracortical lateral interactions in barrel cortex:

1. Primary somatosensory cortex is topographically organized and contains an orderly somatotopic representation of the body surface. This is particularly clear in barrel cortex, where each whisker is anatomically and physiologically linked to a single barrel and the barrel organization preserves neighborhood relationships between whiskers (Woolsey and Van Der Loos, 1970; Hall and Lindholm, 1974; Chapin and Lin, 1990). Preservation of neighborhood relationships in the cortical representation means that lateral interactions can be instantiated by relatively short intracortical connections.
2. It is highly likely that naturalistic whisker stimulation patterns are correlated across whiskers. Specifically, during many behaviors, multiple whiskers are likely to contact surfaces at roughly the same time, potentially leading to simultaneous activation of many neurons in nearby locations in primary somatosensory cortex (Brecht et al., 1997; Sachdev et al., 2001; Jadhav et al., 2009; O'Connor et al., 2010b). This is analogous to the redundancies present in natural visual stimuli, which show a high degree of local correlation structure (Field, 1987). Local intracortical connections are well situated to reduce the redundancy of the cortical response by enhancing response sparseness.
3. There are multiple anatomical substrates for lateral connections. Excitatory spiny stellate cells are synaptically connected to each other, and project to supragranular layers directly above the barrel, which in turn give rise to the widespread lateral connections in supragranular layers (Gottlieb and Keller, 1997; Kim and Ebner, 1999; Reyes and Sakmann, 1999; Staiger et al., 2000; Brecht and Sakmann, 2002a; Feldmeyer et al., 2002; Shepherd and Svoboda, 2005; Egger et al., 2008). There are few direct connections between layer IV barrels (Kim and Ebner, 1999), but there are lateral connections generated by layer V neurons into layer II/III (Larsen and Callaway, 2006). These large-scale lateral connections within barrel field provide a mechanism for lateral interactions between different spatial locations in the barrel field (Kim and Ebner, 1999; Chakrabarti and Alloway, 2006; Larsen and Callaway, 2006; Lee et al., 2008). The lateral interactions generated by pyramidal neurons *in vitro* can be very specific: stimulation of layer II/III pyramidal neurons suppressed activity of layer II/III pyramidal neurons in neighboring columns, facilitated activity of layer V pyramidal cells, and had little effect on neurons in layer IV and VI (Adesnik and Scanziani, 2010). In addition to the excitatory neurons that connect columns, some interneurons also project within multiple columns (Helmstaedter et al., 2009). Their axons can ramify exclusively within a single column, or can project laterally to influence activity in neighboring columns.
4. Single pyramidal neurons in barrel cortex have large multi-whisker receptive fields. The surround receptive fields could at least partially be generated by lateral interactions between barrel columns; the difference in latency to respond to center and surround whiskers could arise from intracortical synaptic delays (Armstrong-James and Fox, 1987; Nicolelis et al., 1995; Brecht and Sakmann, 2002a; Brecht et al., 2003). Whisker stimulation can also suppress firing of neurons in barrel cortex: suppression of activity requires either an increase in intracortical inhibition or a withdrawal of cortical input (Sachdev et al., 2001; Krupa et al., 2004).
5. There is evidence of traveling waves of activity in barrel cortex. Single whisker stimulation evokes depolarization that begins in the corresponding barrel, spreads across the entire extent of barrel cortex and then into adjacent cortical areas (Kleinfeld and Delaney, 1996; Derdikman et al., 2003; Petersen et al., 2003; Ferezou et al., 2006, 2007; Frostig et al., 2008; Davis et al., 2011). The wave is thought to be propagated by lateral connections within barrel cortex and by diffuse connections between adjacent cortical areas (Frostig et al., 2008).

## Intracortical inhibition in barrel cortex

Barrels are unusual cortical cell aggregates in that they contain nearly as many inhibitory as excitatory neurons in the barrel domains. Excitation and inhibition in barrel cortex are balanced, both at rest and in response to whisker stimulation (Hasenstaub et al., 2007; Okun and Lampl, 2008). However, some sensory stimuli can differentially engage excitatory and inhibitory cortical circuits. Both the cortical state (up or down) and the adaptation state of the local cortical inputs can determine the degree to which stimuli engage inhibitory circuits (Erchova et al., 2002; Petersen et al., 2003; Sachdev et al., 2004; Hasenstaub et al., 2007; Heiss et al., 2008). In addition, Gentet and colleagues (2010) reported that while the membrane potential oscillations of interneurons and pyramidal cells can be synchronized, during active whisking, the firing rate of the fast spiking interneurons decreased, the firing rate of the non-fast-spiking interneurons increased and there was no net change in the firing rate of pyramidal neurons. These observations seem to indicate the relationship between ongoing spontaneous activity and stimulus-evoked inhibition and excitation is highly dependent on both behavioral and brain state.

Whisker stimulation activates putative interneurons in rabbit and rodent somatosensory cortex (Simons, 1978; Swadlow, 1989; Brumberg et al., 1996). Fast-spiking interneurons respond to whisker stimuli at short latencies. Interestingly, these interneurons have larger spatial receptive fields than pyramidal neurons (Swadlow, 1991; Bruno and Simons, 2002; Khatri et al., 2004) and are preferentially targeted by thalamocortical inputs (Bruno and Simons, 2002; Cruikshank et al., 2007). In the thalamocortical slice, electrical stimuli reliably elicit action potentials from layer IV interneurons, while the same stimulation protocol is much less effective in evoking spikes from excitatory neurons (Agmon and Connors, 1992; Porter et al., 2001). Finally, in cortical slices, intracortical activation of pyramidal cells recruits somatostatin inhibitory interneurons preferentially (Kapfer et al., 2007).

## Surround suppression in whisker to barrel cortex

Surround suppression in barrel cortex is usually studied by pairing stimulation of the center, or principal, whisker with stimulation of one or more adjacent (surround) whiskers. Though a few studies show that paired whisker stimulation can evoke facilitation (Shimegi et al., 1999, 2000; Ego-Stengel et al., 2005; Hirata and Castro-Alamancos, 2008), the preponderance of studies show that co-stimulation or stimulation of one whisker followed by the other, suppresses responses to the surround or second whisker. The cortical response to repeated deflections of a single whisker is suppressed when the interval between deflections is shorter than 100 ms (Fanselow and Nicolelis, 1999; Chung et al., 2002; Arabzadeh et al., 2003; Garabedian et al., 2003; Melzer et al., 2006; Drew and Feldman, 2007; Khatri and Simons, 2007; Sanchez-Jimenez et al., 2009; Boloori et al., 2010; Stuttgen and Schwarz, 2010). Similarly, whisker deflection attenuates the response to a second whisker for a period of 10–200 ms, with maximal suppression at 20 ms (Simons, 1985; Simons and Carvell, 1989; Brumberg et al., 1996; Kleinfeld and Delaney, 1996; Mirabella et al., 2001; Higley and Contreras, 2003; Ego-Stengel et al., 2005; Higley and Contreras, 2005; Boloori and Stanley, 2006; Webber and Stanley, 2006; Drew and Feldman, 2007; Higley and Contreras, 2007). Although surround suppression in barrel cortex is well-established and characterized, the underlying mechanisms are not yet understood. The original studies of surround inhibition by Carvell and Simons (1990) indicated that there was less surround suppression in thalamic neurons compared to cortical neurons, suggesting surround suppression was at least partially a cortical phenomenon. Brumberg and colleagues (1996), using a different stimulation protocol, confirmed this finding and reported surround suppression was restricted to regular spiking pyramidal neurons. More recent studies have provided evidence of subcortical mechanisms. Higley and Contreras (2003, 2005, 2007), using intracellular and extracellular methods (in combination with current source density analysis), demonstrated that surround suppression in S1 was primarily associated with a withdrawal of excitation, based on observed changes in the pattern and strength of subcortical inputs to S1. They also found that cortical surround suppression persisted following application of the GABA agonist muscimol. This work also showed unambiguously that multi-whisker stimulation could elicit robust surround suppression in thalamus. The results from Higley and Contreras (2003, 2005, 2007), as well as Deschenes and colleagues (Varga et al., 2002; Lavallee and Deschenes, 2004; Timofeeva et al., 2004), are most consistent with a subcortical origin for surround suppression in barrel cortex.

At this time, there is little reason to believe that either subcortical or intracortical inhibition is the sole source of surround suppression in barrel cortex. Instead, it seems more likely that lateral interactions at multiple levels (brainstem, thalamus, and perhaps cortex) all contribute to surround suppression. There are several potential differences between experiments that support intracortical inhibition (Brumberg et al., 1996) and those that support subcortical mechanisms (Higley and Contreras, 2003, 2006, 2007). The key differences between these studies may be the anesthetic state or the stimuli. Brumberg and colleagues used spectrotemporally complex stimuli, while Higley and Contreras used a more traditional ramp and hold stimulus. As in V1, stimulus properties could alter patterns of inhibition, with more spectrally complex stimuli evoking more dominant intracortical inhibition. Again, as in V1, these questions need to be addressed by future research.

## Connecting surround suppression, lateral inhibition, and sparse coding

Almost by definition, surround suppression depends on lateral inhibitory processes. However, as should be clear from the above discussion, the source of this inhibition, specifically whether or not it has an intracortical origin, remains a matter of substantial debate in both visual and barrel cortex. It is possible that discrepancies between the different studies of visual and barrel cortex can be partially, or even completely, accounted for by differences in stimulation protocols; however, this hypothesis has yet to be tested experimentally. The use of optogenetic methods to activate well-defined, highly localized inhibitory circuits in visual and barrel cortex during sensory stimulation may help elucidate the role of different inhibitory circuits in generating surround suppression.

There is a growing body of experimental and theoretical evidence suggesting that surround interactions are critical for generating sparse sensory responses in cortex. Some of the established computational models for generating sparse sensory representations are likely to also reduce spontaneous activity (Olshausen and Field, 1996). In theory, low spontaneous activity and weak responses could simply reflect low levels of intrinsic activity in neocortical neurons. Alternatively, low spontaneous and response levels could be due to intracortical or subcortical surround interactions. It is worth noting that few cortical neurons are intrinsically spontaneously active, i.e., most cortical neurons do not spike once synaptic transmission is pharmacologically blocked within cortex (Sanchez-Vives and McCormick, 2000; Compte et al., 2003; Cossart et al., 2003). Neocortical neurons and circuits might be unique in their relative scarcity of intrinsically active neurons: in the basal ganglia (pallidal, nigral, and interneurons in striatum), cerebellum (purkinje cells and inferior olive neurons) and thalamus whole classes of neurons are spontaneously active in the absence of synaptic input. It remains to be determined whether low spontaneous activity in cortex reflects cellular or network properties.

The original rationale for considering sparse codes was based on the idea that a sensory representation where neurons are generally silent and spike only occasionally, in response to a limited number of stimuli, would be more metabolically efficient, given the metabolic cost of repolarizing cell membranes following action potentials (Barlow, 1961; Levy and Baxter, 1996; Treves et al., 1999; Attwell and Laughlin, 2001; Lennie, 2003). The alternative to a sparse code is a dense code, in which many or all neurons are essentially continuously active, which results in a high mean firing rate across the population and is metabolically more expensive to sustain. Response sparseness and low spontaneous activity are not identical, although some cortical models that instantiate a sparse code might also result in low spontaneous activity. Similarly, lifetime sparseness, where neurons are generally silent and respond vigorously to only a few stimuli, and population sparseness, where only a few neurons in the population are active at any given time (see Wolfe et al., 2010; Willmore et al., 2011) should not be confused.

A number of studies in somatosensory cortex have reported both extremely low spontaneous activity and high trial-to-trial response variability (Brecht et al., 2003; Kerr et al., 2007; Huber et al., 2008; Poulet and Petersen, 2008; Jadhav et al., 2009; Gentet et al., 2010; O'Connor et al., 2010b); other recent studies have shown that barrel cortical neurons can have high spontaneous activity levels (8 Hz) that can be further increased (up to 30 Hz) when whiskers come in contact with rough surfaces (Vijayan et al., 2010). Low spontaneous activity could very well arise from an Olshausen and Field-like network that depends on mutual inhibition to generate sparseness. As noted above, many of the studies that address sparseness in S1 focus on the low mean rate definition of sparseness, while V1 studies have focused primarily on lifetime or population sparseness. Lifetime and population sparseness provide insights into the nature and efficiency of the neural code. A sparse representation is one that that eliminates the spatial and temporal redundancies intrinsic to natural visual stimuli. In barrel cortex, more work is needed to characterize the defining properties and intrinsic redundancy that arise during normal whisking behavior. Understanding the degree to which stimulus variation affects lifetime and population sparseness will facilitate direct comparisons with work in the visual system.

In visual cortex, the relationship between surround suppression and sparse coding was explicitly tested in the studies using naturalistic whole-field stimuli (Vinje and Gallant, 2000). Cortical neurons become more selective during naturalistic whole-field stimulation and this increased selectivity appears to be directly related to increased suppressive surround interactions. To date there have been no studies of natural whisking behavior sufficiently detailed to be able to characterize the spatiotemporal correlations of whisker inputs during natural behavior. However, the physical organization of the whisker system makes it highly likely that are spatiotemporal correlations in the sensory input. The few studies in barrel cortex that have measured the effects of surround suppression on selectivity (e.g., Jacob et al., 2008) suggest surround suppression enhances selectivity, which could result in increased response sparseness. However, to the best of our knowledge, there have been no studies measuring the lifetime response sparseness of single neurons in barrel cortex in the way done by Vinje and Gallant. It is not yet known if spatiotemporal correlations in whisker inputs is, or even can be, removed at the level of barrel cortex. In the whisker system, spatiotemporal correlations, perhaps driven by simultaneous object contact, could be ethologically relevant signals; decorrelation could potentially eliminate important sensory signals. To close the loop and answer some of the questions raised here, we need to better understand the responses of neurons to multiple whisker stimuli when the stimuli are both correlated and uncorrelated across whiskers.

It is important to note that sparseness is not without costs—while a sparse code may facilitate downstream decoding, transmit more information about the stimuli, or be metabolically efficient, it necessarily requires more neurons than a dense or intermediately sparse code (think grandmother cells!). Biological systems must balance the potential gains obtained from sparse coding against the cost of building more neurons.

A general rule for both, or even all, sensory systems may be that the spatial and temporal dynamics of natural sensory stimulation invariably activate both the center and surround components of the receptive field in most sensory neurons. The lateral interactions, at many synaptic levels, engaged by spatially extensive stimuli are likely to increase the sparseness of the neural code and increase selectivity in sensory cortex.

### Conflict of interest statement

The authors declare that the research was conducted in the absence of any commercial or financial relationships that could be construed as a potential conflict of interest.
